# Transglutaminase 2 maintains a colorectal cancer stem phenotype by regulating epithelial-mesenchymal transition

**DOI:** 10.18632/oncotarget.27062

**Published:** 2019-07-16

**Authors:** Oluseyi Ayinde, Zhuo Wang, Giulia Pinton, Laura Moro, Martin Griffin

**Affiliations:** ^1^Department of Biology and Biomedical Science, School of Life and Health Sciences, Aston University, Aston Triangle, Birmingham B4 7ET, United Kingdom; ^2^Department of Pharmaceutical Sciences, Università degli Studi del Piemonte Orientale, Novara 28100, Italy

**Keywords:** transglutaminase 2, colorectal cancer, epithelial-mesenchymal transition, cancer Stem Cells, β-catenin

## Abstract

Transglutaminase 2 (TG2), a multifunctional protein, is reported in regulating the cancer stem cell (CSC) phenotype in various cancers. Our previous work suggested the link between TG2 and Epithelial-Mesenchymal Transition (EMT) in colorectal cancer (CRC). Here we demonstrate the importance of TG2 in CSC development in human CRC cell lines HCT116 and SW620. CRC spheroid cells showed increased CSC characteristics over their monolayer cells with increased expression of CD44 and over expression of Oct3/4, Sox2 and Nanog. They also showed increased EMT and invasiveness, and enhanced expression of TG2. TG2 inhibition by its selective inhibitor 1-155 reduced both spheroid formation and invasive potential of the spheroid cells. β-catenin, a mediator of stem cell maintenance, was overexpressed in the spheroid cells and could be attenuated by TG2 inhibition. Spheroid cells possessed increased angiogenesis stimulating ability via overexpression of Vascular Endothelial Growth Factor (VEGF). Increased VEGF was present in the culture media from spheroid cells when compared to monolayer cultures which could be reduced by selective inhibition by 1-155. Stemness and malignancy in the colorectal spheroid cells was associated with increased TG2, EMT, β-catenin and VEGF. Here we demonstrate that inhibiting TG2 reduces both stemness and angiogenic stimulating activity in CRC.

## INTRODUCTION

There is a growing body of evidence showing that the formation and seeding of circulating tumour cells are highly dependent on a subpopulation of tumour cells with self-renewal potential and ability to differentiate into diverse tumour populations, this subpopulation of cells is called the cancer stem cells (CSCs) that can repopulate a tumour or seed a metastasis [1]. In colorectal cancer (CRC), patient survival depends on the stage of the disease, with metastasis accounting for poor patient prognosis. More recently, cancer researchers have become aware of the existence of colorectal CSCs, which possess self-renewing capabilities and essentially have the potential to acquire the many mutations that result in a cancerous cell [2]. These CSCs form the hierarchy of tumours and possess metastatic and drug-resistant phenotypes [3]. Wei *et al* (2012) have also shown that CSCs can sustain carcinogenesis, angiogenesis, metastasis and recurrence of CRC after remission [4].

In cancer, many of the embryonic and wound healing processes are subverted for pathological gains, one of which is Epithelial-Mesenchymal Transition (EMT), a process which enables epithelial cells to gain a mesenchymal-like phenotype [5]. Various studies are now providing evidence that EMT process is important in the development and acquisition of a CSC phenotype in various epithelial cancers [6, 7].

Transglutaminase 2 (TG2), a multifunctional enzyme associated with pro-inflammatory responses and wound healing [8], has been reported to regulate EMT in various cancerous and fibrotic conditions [9, 10]. In recent times, TG2 expression has been reported to be increased in CSC-like enriched populations in ovarian cancer [11], breast cancer [12], squamous carcinomas [13] and mesotheliomas [14]. In addition β-catenin, an oncogenic protein that is widely upregulated in ovarian [15], gastric and CRC [16], has been shown to be important in the formation of CSC by targeting and activating the transcription of a number of genes which play an important role in maintenance of intestinal CSC [17]. Previous studies in CRC have shown that TG2 may potentiate nuclear accumulation of β-catenin in cancer cells [15, 18].

In this study, we show that by enrichment of a CSC population *in vitro*, TG2 is upregulated and that TG2 in association with β-catenin plays a role in the regulation of EMT and in the induction of the colorectal CSC phenotype, including tumour angiogenesis.

## RESULTS

### CD44 and transcription markers of stem cells are upregulated in spheroid cells

**Figure 1:**
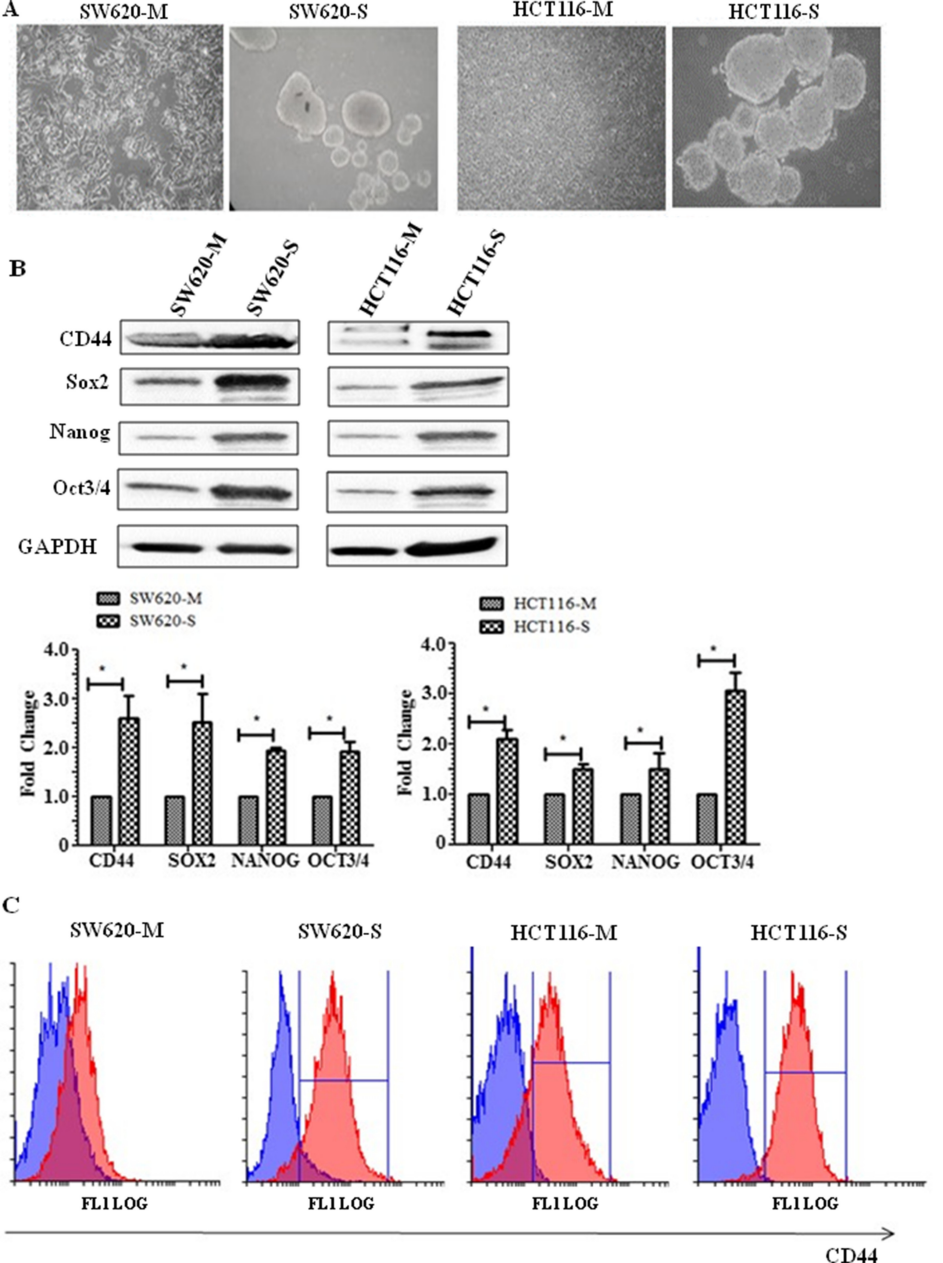
Spheroid cells display stem-like properties. **(A)** Representative phase-contrast microscope images (20× objective) of HCT116 monolayer (M), HCT116 spheres (S), SW620 monolayer and SW620 spheres. **(B)** Representative Western blot showing cell surface protein CD44, and transcription markers associated with stem cells Sox2, Nanog, and Oct3/4. The graph shows mean densitometry values ± S.D. compared to controls (taken as 1.0) * indicates significantly different (p<0.05). n=3. **(C)** Flow cytometry analysis of CD44+ sub-populations of SW620 and HCT116 monolayer and spheroids. Cells were stained with anti-human CD44- phycoerythrin-(PE) conjugated mouse antibody. Isotype-matched human antibodies served as controls. Isotype represented in blue and CD44 positive cells represented by the red graph. n=3.

An *in vitro* spheroid formation assay has been widely documented as a means to selectively grow colon cancer cells with stem-like characteristics able to initiate tumour growth in immunodeficient mice and was used to enrich CRC cells with stem cell-like properties [19]. In Figure 1A, the morphology of monolayer and spheroids is shown. The tumour spheroid is composed of cellular aggregates which have contracted to form a compact spheroid structure. In whole cell lysates, the CD44 cell surface protein marker for CSCs shows a significant increase in expression in the SW620 and HCT116 spheroid cells (SW620-S and HCT116-S, respectively) compared to their parental monolayer cells SW620-M and HCT116-M (Figure 1B). This finding was confirmed by flow cytometry that measured the cell surface expression of CD44 (Figure 1C). Western blotting was also used to detect the expression levels of transcription factors that upregulate cell stemness in both SW620 and HCT116 cells. Figure 1B shows that the expression of transcription factors Sox2, Nanog and Oct3/4 were significantly increased in the spheroid cells compared to the monolayer cells (Figure 1B).

### TG2 and EMT are upregulated in CRC

**Figure 2:**
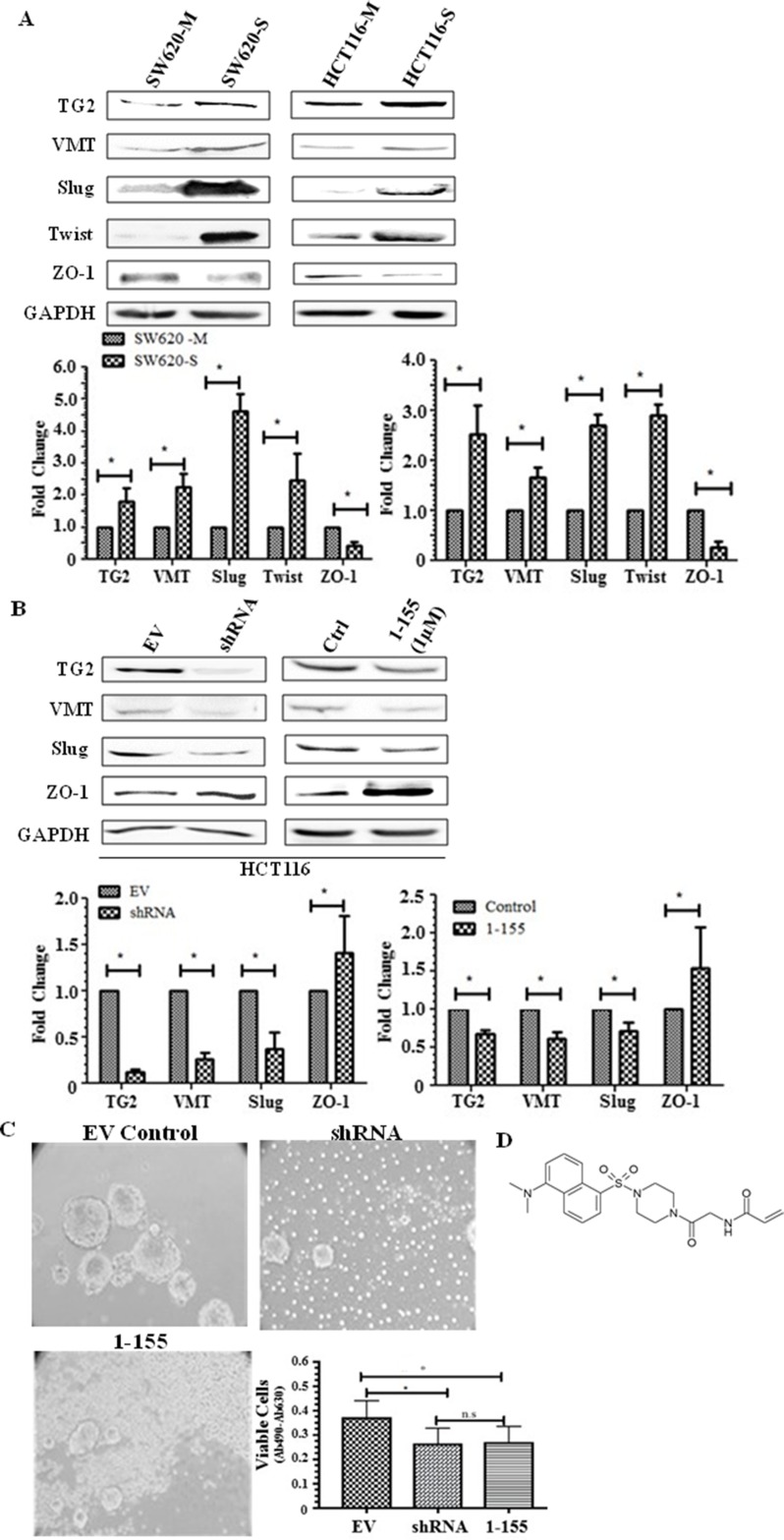
TG2 and EMT is upregulated in spheroid cells with stem cell-like properties. **(A)** Representative Western blot of whole cell lysates of parental adherent monolayer cells and spheroid cells from SW620 and HCT116 cells showing expression of TG2 and vimentin, transcription markers of EMT, Slug and Twist, and epithelial tight junction protein ZO-1. The graph shows mean densitometry values ± S.D. compared to controls (taken as 1.0). *, indicates significantly different (p<0.05). n=3. **(B)** The effect of TG2 knockdown or inhibition on EMT markers in HCT116 cells. Western blots of TG2, mesenchymal marker vimentin, and the transcription factor of EMT, Slug and expression of tight junction protein ZO-1 in whole cell lysates of TG2 knockdown cells and cells treated with TG2 selective cell-permeable inhibitor 1-155 (1μM). EV are cells containing empty vector. Ctrl represents vehicle control. The graph shows mean densitometry values ± S.D. compared to controls (taken as 1.0). * indicates significantly different (p<0.05). **(C)** HCT116 cells transduced with TG2 shRNA or incubated with TG2 inhibitor 1-155 reduces the enrichment of spheroids over 10 days as shown in the images captured using phase contrast microscopy. Cell viability shown in the graph was undertaken as described in the Materials and Methods. * indicates significantly different (p<0.05). **(D)** shows the structure of TG2 inhibitor 1-155.

Following the characterisation and validation of the spheroid cells as possessing stem cell markers, the spheroid cells were characterised for TG2 expression in the whole cell lysates using Western blotting. Figure 2A indicates a significant increase in TG2 expression in spheroid cells, SW620-S and HCT116-S compared to SW620 and HCT116 monolayer cells. A significant increase in the expression of a mesenchymal marker vimentin, while a significant decrease in an epithelial cell tight junction protein ZO-1, was detected in the spheroids of both HCT116 and SW620, compared to their respective monolayer counterparts. Additionally, to confirm the increase of EMT in the spheroids, the expression of transcription factors that regulate EMT were also determined by Western blotting which showed that both Slug and Twist were significantly increased (Figure 2A). We previously showed the importance of TG2 in EMT in SW620 cells using TG2 knockdown or pharmacological inhibition [18]. Figure 2B shows that knockdown of TG2 expression via a shRNA specific for human TG2 in HCT116 led to a significant reduction in the expression of EMT marker vimentin, and a reduction in the expression of EMT transcription factor Slug. In addition, ZO-1, an epithelial tight junction marker, was seen to be significantly increased following TG2 knockdown by shRNA. Figure 2B shows that our TG2-selective and cell permeable inhibitor 1-155 (1 µM for 48 h) was able to reverse the EMT in HCT116 cells. In line with TG2 inhibition leading to a reversal of EMT in both SW620 and HCT116 cells, inhibition of TG2 by 1-155 or pre knockdown of TG2 by shRNA in monolayer cells can also lead to a reduction in spheroid formation in HCT116 cells (Figure 2C) as previously demonstrated for SW620 cells [18]. The structure of the selective TG2 inhibitor 1-155 is shown in Figure 2D.

The effect of the empty vector (EV) and the vector containing TG2 shRNA was investigated in Supplementary Figure 1A, with expression of TG2 and EMT marker vimentin unaffected by the empty vector transduction when compared to wild type (wt) cells. Supplementary Figure 1B shows that TG2 inhibition by 1-155 does not affect the cell viability of HCT116 cells as measured by Sodium 3’-[1-(phenylamino-carbonyl)-3,4-tetrazolium]-bis (4-methoxy-6-nitro) benzene sulfonic acid hydrate (XTT) over a 48 h treatment period.

### Colorectal stem-like cells display an invasive phenotype *in vitro*

**Figure 3:**
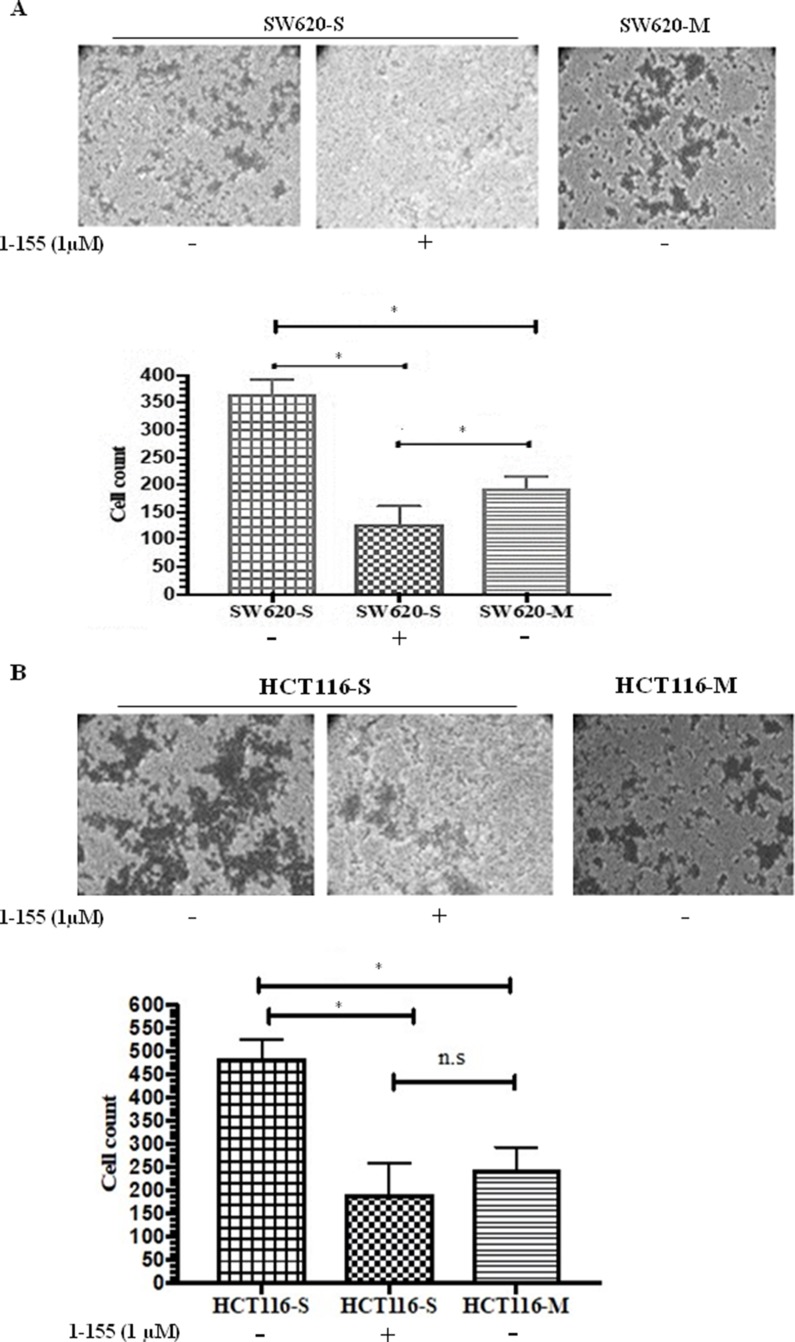
TG2 inhibition reduces invasiveness of CRC spheroid cells with stem cell properties. **(A)** Cell invasion of SW620 spheroid cells and monolayer cells **(B)** Cell invasion of HCT116 spheroid cells and monolayer cells. Representative images of crystal violet stained cells that have invaded collagen IV in the transwell inserts. Images were captured using phase contrast microscopy (10X objective). The graphs show the cell count from each group. For 1-155 (1μM) treated cells, cells were pre-treated with 1-155 for 16h prior to seeding cells and were further treated with 1-155 (1μM) after cell seeding for the duration of the experiment. n=3. * Indicates significantly different (p<0.05). N.S-not significant (P>0.05)

An increased mesenchymal phenotype is found in the spheroids enabling these cells to mediate tissue invasion and metastasis [20]. We next attempted to compare the invasive properties of spheroid cells with stem cell-like properties against the parental monolayer cells, by utilising an ECM Transwell chamber invasion assay. As shown in Figure 3A and 3B, both SW620 and HCT116 spheroid cells demonstrated a significantly increased invasion in the collagen IV-coated Transwell chambers compared to their monolayer counterparts. Incubation of the spheroid cells with the TG2 selective inhibitor 1-155 (1 μM) significantly reduced the invasive potential of the spheroid cells isolated from both SW620 and HCT116.

### Stem cell regulator β-catenin is upregulated in CRC stem cells

**Figure 4:**
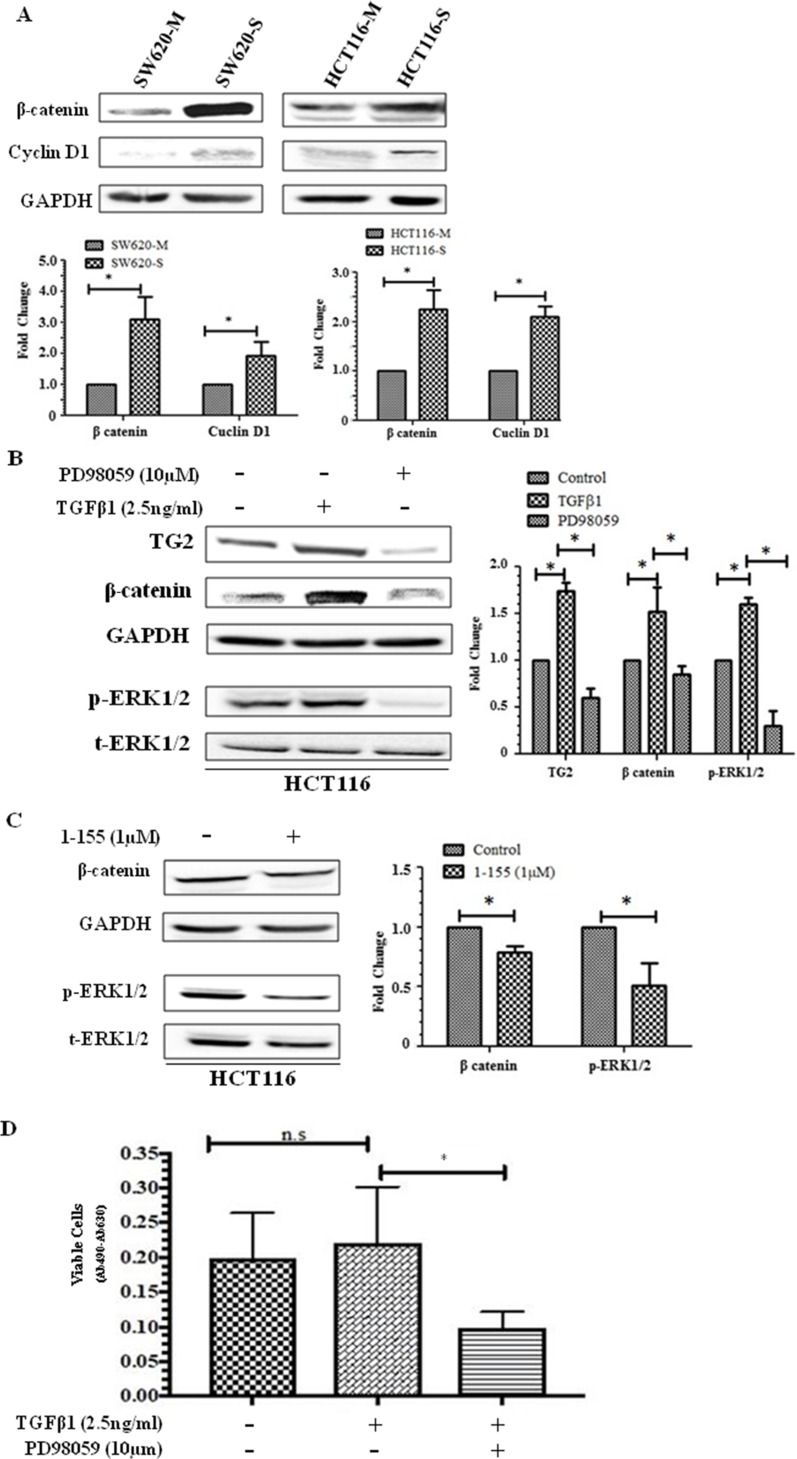
Importance of β-catenin in CSC spheroid formation. **(A)** Representative Western blot of monolayer and spheroid cells from SW620 and HCT116 detecting the presence β-catenin and cyclin D1 expression in whole cell lysate. The graph shows mean densitometry values ± S.D. compared to controls (taken as 1.0) following normalising densitometry values ± against GAPDH. n=3. * indicates significantly different (p<0.05) **(B)** ERK activation increases β-catenin expression in HCT116 cells. Western blot of TG2, β-catenin and ERK activation in whole cell lysates of HCT116 cells treated with human recombinant TGFβ1 (2.5 ng/ml) and ERK inhibitor PD98059 (10 μM) for 48h. The graph shows mean densitometry values ± S.D. compared to controls (taken as 1.0) following normalising densitometry values ± S.D. against GAPDH. n=3. * indicates significantly different (p<0.05). **(C)** TG2 inhibition reduces ERK activation and β-catenin expression in HCT116 cells. Representative Western blot of TG2, β-catenin and ERK1/2 activation (p-ERK) in whole cell lysates of HCT116 monolayer cells following treatment with TG2 inhibitor 1-155 (1 µM) for 48 h. The graph shows mean densitometry values ± S.D. compared to controls (taken as 1.0) following normalising densitometry values against GAPDH. n=3. * indicates significantly different (p<0.05). **(D)** ERK1/2 inhibition reduces spheroid forming potential of HCT116 cells. HCT116 cells were seeded in spheroid forming media containing PD98059 (10 μM) or TGFβ1 (2.5 ng/ml) for 10 days and cell viability was performed using XTT. Control cells were treated with vehicle alone. n=3. *, significantly different from PD98059 treated cells. N.S, not significant.

β-catenin is a regulator of stem cell maintenance and differentiation and is upregulated in CSCs [21]. In Figure 4A, spheroid cells exhibit over a 2-fold increase in β-catenin expression compared to monolayer cells and a significantly increased expression of its target gene cyclin D1 in spheroid cells suggesting that there is increased β-catenin nuclear transcription activity in these cells. TG2 has been reported to regulate β-catenin function in ovarian cancer cells [15] and SW620 CRCs [18]. TGFβ1 at 2.5 ng/ml was found to induce TG2 and β-catenin in HCT116 cells, while ERK1/2 inhibition by PD98059 (10 µM) significantly reduced the expression of TG2 and reduced the β-catenin expression levels back to pre-TGFβ1 stimulation (Figure 4B). Furthermore, TG2 inhibition also led to a small but significant reduction in ERK1/2 activation and β-catenin expression. The effect of TGFβ1 on spheroid formation in HCT116 cells indicated no significant effect on cell viability (Figure 4D). However, ERK1/2 inhibition by P98059 significantly reduced the spheroid forming potential of HCT116 cells as determined by XTT, after 10 days of spheroid formation (Figure 4D). In contrast, we have previously shown that neither TGFβ1 treatment nor its inactivating antibody had any significant impact on the EMT status of SW620 cells, and that the basal protein expression of ERK1/2 in SW620 cells is already very low [18].

### Expression of VEGF is increased in spheroid cells giving an enhanced angiogenic effect on endothelial cells

**Figure 5:**
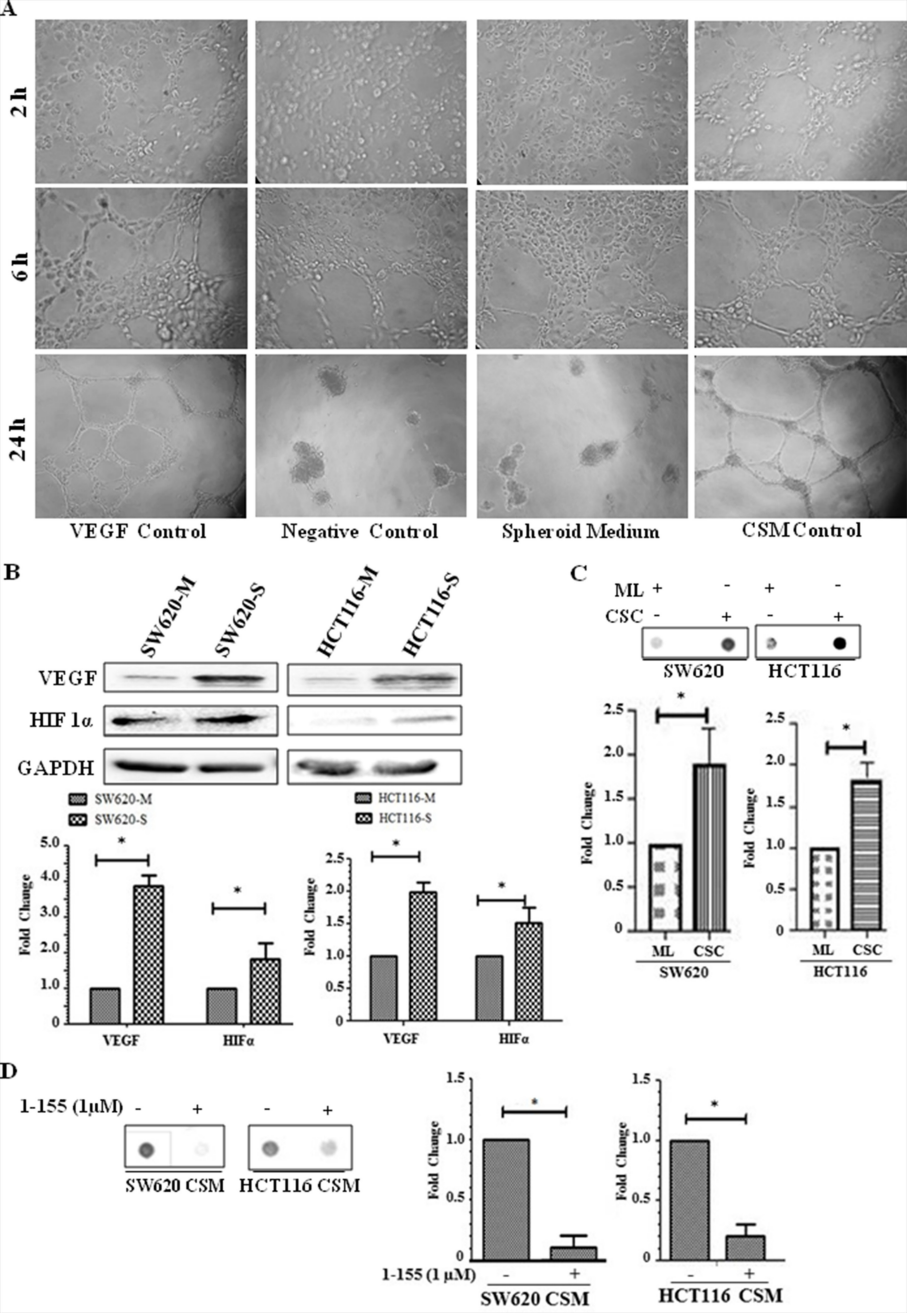
Spheroids containing CRC stem-like cells induce angiogenesis *in vitro*. **(A)** HUVEC cells (3000 cells/well) were seeded into 96-well plates containing Matrigel and induced to form cord like structures. HUVEC cells were treated with 10 ng/ml rhVEGF which served as positive control, while DMEM/F12 basal medium was used as the negative control. Spheroid cell media without cell growth served as a control for the spheroid cell conditioned media (SCM) after spheroid cell growth for 18 h. Representative images are shown from cells treated for 24 h and phase contrast images using a 10× objective were taken at 2, 6 and 24 h periods. n=2. **(B)** Western Blot of monolayer and spheroid cells from SW620 and HCT116 detecting the presence VEGF and HIF1α expression in whole cell lysate. The bar chart shows mean densitometry values ± S.D. compared to controls (taken as 1.0) following normalising of protein loading using GAPDH. n=3. * indicates significantly different (p<0.05). **(C)** Dot blot of monolayer (ML) and spheroid cells (CSC) from SW620 and HCT116 detecting the presence of VEGF in cell culture medium as described in the Materials and Methods. The graph shows mean densitometry values ± S.D. for VEGF normalised against media from monolayer cells (taken as 1.0). n=3. * indicates significantly different (p<0.05). **(D)** Representative Dot blot of VEGF present in conditioned media of spheroid cells (CSM) from SW620 and HCT116 cultured in the presence or absence of 1-155 as described in the Materials and Methods. The graph shows mean densitometry values ± S.D. for VEGF normalised against the vehicle control (taken as 1.0). n=3. * indicates significantly different (p <0.05).

Tumours originating from CSCs are more vascular compared to tumours from monolayer cells [4]. In Figure 5A, Human Umbilical Vein Endothelial Cells (HUVEC) cells seeded on Matrigel were treated with rhVEGF or conditioned media from the cultured SW620 spheroid cells over a 24-hour period. HUVECs treated with conditioned media for 6 h started to form cords and by 24 h endothelial cords were observed in both rhVEGF and condition media treated HUVEC cells. In the unconditioned spheroid cell media control and the minus VEGF control, endothelial cord formation was not evident at this time period. The spheroids were next characterised for their expression of VEGF which indicated increased cellular expression of VEGF, and also HIF1α when compared to their respective monolayer cells (Figure 5B). Dot blot analysis showed VEGF was detectable in both the conditioned media from the cancer enriched CSCs isolated from the spheroids and the corresponding monolayer cultures, but in keeping with the increase in VEGF expression, in the spheroids, increased levels of VEGF were found in the conditioned media from the spheroid derived cells (Figure 5C). To confirm a potential relationship between TG2 and the presence of VEGF in the cell culture media, SW620 and HCT116 cells isolated from the spheroids were treated with 1-155. Reduced levels of VEGF were detected in the presence of 1-155, compared to the controls. (Figure 5D).

## DISCUSSION

The use of *in vitro* spheroids in culture has been widely shown and used as a means to isolate, enrich, maintain or expand potential CSC subpopulations from various types of cancers [7, 22-24], and these CSC populations show stem cell-like properties and expression of CSC markers. Here, we characterised the spheroids obtained from highly invasive CRC cells SW620 and HCT116 for the expression of the stem cell marker CD44. We show that CD44 was upregulated in the spheroids cultured from both cells as assessed via flow cytometry and Western blotting. In addition, we also show that transcription factors of stemness, including Oct3/4, Nanog, and Sox2 [25], were highly expressed in the SW620 and HCT116 spheroid cells, compared to the parental monolayer cells.

Various studies are now providing evidence of a role for EMT in the acquisition of the CSC phenotype in CRC cells [20, 26, 27]. It has been proposed that while EMT is necessary for cells to dissociate, evade and disseminate (metastatise), the last metastatic step involves the growth of micrometastases into macroscopic metastases, and requires cells with self-renewal capacity and proliferative potential [28].

In our current study, we show that the EMT phenotype is heightened in spheroids of SW620 and HCT116 cells with a stem cell-like phenotype. While there is still a host of hypotheses concerning the origins of CSCs, numerous studies have suggested that EMT may facilitate CSC formation. Fan *et al* (2012) showed that transcription factors of EMT (Snail and Twist) confer a CSC phenotype by inducing the expression of pluripotency maintaining transcription factors associated with stem cells, such as Oct3/4, Sox2, Nanog in CRC cell lines HT29 and HCT116 [27]. Here, we confirmed the increased expression of EMT transcription factors Slug and Twist in both SW620 and HCT116 spheroid cells, compared to monolayer cells, which coincides with the expression of Oct3/4, Nanog and Sox2. Importantly, we demonstrated that the expression of TG2 was upregulated in the spheroids from both SW620 and HCT116 in comparison to the cells cultured in monolayers. Similar findings have been reported in ovarian, breast and squamous carcinomas [29]. Our previous study showed that selective TG2 inhibition reduced the spheroid forming capacity of SW620 colorectal cells [18], with similar observations made in ovarian [11], breast [30] and squamous carcinomas [11, 12, 22]. In this paper, we show that both TG2 shRNA and TG2 inhibition by its selective small molecule inhibitor 1-155 developed in our group [31] can reduce spheroid formation in HCT116 cells. This suggests a role for TG2 in CSC formation *in vitro*. We have also reported that TG2 may induce EMT in CRCs and regulate the expression of both Slug and Twist. The mechanism by which TG2 mediates EMT could be cell type specific and also dependent on its degree of progression. In our previous report, we showed that in non-metastatic primary CRC cells (RKO and SW480), TGFβ1 expression, release and signalling either through the canonical Smad signalling or via regulating Mitogen-activated protein kinases (MAPK) signalling was regulating EMT[18]. In contrast in the lymph metastatic cell line SW620, TG2 regulated EMT by facilitating nuclear translocation of β-catenin and demonstrated that the highly TG2-specific small molecule inhibitor 1-155 or silencing of TG2 by shRNA attenuated the EMT phenotype [18]. In this present study, we confirm that TG2 inhibition by 1-155 or TG2 knockdown by shRNA in HCT116 monolayer cells reduces EMT. As TG2 is increased in CSCs it is very plausible that increased TG2 and EMT facilitates the stem cell formation in these CRCs in an *in vitro* tumour spheroid formation assay.

β-catenin is an oncoprotein whose deregulation is associated with CRCs [32] and plays an important role in the maintenance of intestinal stem cells. Nuclear β-catenin signalling in embryonic stem cells, depending on its binding partner can maintain pluripotency or play a role in the lineage decision/commitment process [21]. β-catenin has been shown to up-regulate pluripotency factors, e.g. Oct3/4, Nanog, and Sox2 [17] that are upregulated in the spheroids of both SW620 and HCT116 cells. Therefore, it is not surprising that β-catenin is also upregulated in these spheroid cells. We previously showed that TG2 influenced β-catenin accumulation in the nuclei of SW620 cells, and this may involve an extracellular or intracellular role, as TG2 was also found to complex with LRP5 a co-receptor of Frizzle in the potentiation of Wnt signalling. The observation of transglutaminase involvement in Wnt signalling in ovarian cancer cells has also been reported by Condello *et al* [33]. In addition, intracellular TG2 was shown to complex with β-catenin, limiting β-catenin’s interaction with ubiquitin [18]. In the HCT116 and SW620 spheroid cells, both TG2 and β-catenin were upregulated, compared to monolayer cells, suggesting a role for TG2 in maintaining the CRC phenotype by facilitating β-catenin translocation into the nucleus. However, the upstream pathways regulating involvement of TG2 seem to differ in HCT116 and in SW620. In the HCT116 cells our data suggests a potential role for TG2 in regulating the stabilisation of β-catenin by involvement of the MAPK pathway. Indeed, it has been suggested that ERK1/2 may activate the Wnt/β-catenin signalling [34]. In addition, Horst *et al* (2012) showed that ERK1/2 activation resulted in nuclear accumulation of β-catenin in clinical human CRC cells, and concluded that MAPK signalling contributes significantly in determining the impact of Wnt activity on the stem cell phenotype in CRC cells [35]. In this current study, we demonstrate ERK1/2 inhibition by its inhibitor PD98059 perturbed spheroid formation *in vitro* and TG2 inhibition by its inhibitor 1-155 led to a reduction in ERK1/2 phosphorylation which correlated with decreased β-catenin expression. Taken together this suggests that involvement of TG2 in β-catenin nuclear accumulation may be necessary for maintaining EMT and cancer stemness. To further confirm the nuclear activity of β-catenin, the expression of cyclin D1, a protein that is transcribed following β-catenin nuclear activity [36, 37], was evaluated. In this study, we show that cyclin D1 expression is higher in spheroid cells compared to monolayer cells, suggesting an increased nuclear transcriptional activity for β-catenin.

At this stage it is difficult to draw a definitive conclusion regarding the exact biochemical mechanism that regulates the expression of VEGF in the monolayer cells of both SW620 and HCT116 and which significantly increases in CSC rich spheroids. It has been reported that both EMT and VEGF production is linked to tumour progression and CSC formation and correlations have been made between EMT markers and VEGF expression in a number of different tumours [38, 39]. Wei *et al* (2012) reported that CSCs maintain angiogenesis and there are considerable reports also confirming β-catenin regulation of angiogenesis [4]. According to Zhang *et al* (2001), there are seven β-catenin/Tcf binding sites on the VEGF A (VEGFA) gene promoter [40]. In mice heterozygous for the multiple intestinal neoplasia gene (Min), there was increased redistribution of VEGF in proximity to those cells expressing nuclear β-catenin with a corresponding increase in vessel density [41]. It was further reported that in HeLa cells transfected with constructively active β-catenin, there was an up-regulated VEGF promoter activity by about 4.5-fold [40]. In Figure 5C, spheroid cells rich in CSCs overexpressing β-catenin demonstrate increased expression of VEGF and show increased levels of VEGF found in the cell culture medium compared to monolayer cells in both SW620 and HCT116 cells. Furthermore, HIF1α, a transcription factor also involved in angiogenesis was also elevated in spheroid cells derived from SW620 and HCT116. Interestingly, treatment of cells with the selective TG2 inhibitor 1-155 reduced the amount of VEGF found in the cell growth medium from the spheroid cells. Taken together, an important role for TG2 in the crosstalk between colorectal CSCs and endothelial cells can be proposed in which TG2 is important for both EMT and nuclear β-catenin accumulation which leads to increased VEGF expression. In both EMT and β-catenin accumulation, TG2 appears to be an important player which may explain why TG2 inhibition results in a reduction of VEGF in the culture medium. It is also possible that extracellular TG2 released by CSCs may increase the bio-availability of VEGF by facilitating its matrix binding as suggested by Wang *et al* in endothelial cell tubule formation [42]. The neovascularisation of CSCs in tumours then potentiates tumour growth and metastasis by favouring extravasation.

In summary, our data show that TG2 expression is upregulated in colorectal CSCs and that TG2 plays a role in maintaining the CSC phenotype by inducing EMT and/or by fostering the nuclear activity of β-catenin. We also show that TG2 is important in the accumulation of VEGF in the extracellular environment of CRC cells encouraging endothelial cell tubule formation leading to tumour vascularisation and subsequent tumour progression. Since binding of the cell permeable irreversible inhibitor 1-155 to TG2 fixes the enzyme in its open confirmation blocking both transamidation and GTP/GDP binding [31], at this stage we cannot rule out either of these functions in the development of the CSC phenotype. However, our result indicate that the binding of TG2 to a selective small molecule irreversible inhibitor reduces EMT and attenuates the invasive and angiogenesis stimulating activity of CSCs, strongly suggesting TG2 is an ideal therapeutic target for inhibiting the CSC phenotype in highly aggressive CRCs.

## MATERIALS AND METHODS

### Reagents and antibodies

Peptidomimetic cell-permeable TG2 selective inhibitor 1-155 was synthesized at Aston University [31]. ERK1/2 inhibitor PD98059 and recombinant human TGFβ1 (rhTGFβ1) were purchased from CST (London, UK) and R&D Systems (Bio-Techne Ltd, Abingdon, UK), respectively. Antibodies for Western blot detection of Twist, Slug, β-catenin, Nanog, Oct3/4, Sox2, CD44, VEGF, GADPH, vimentin, total ERK1/2, and phosphorylated ERK1/2 (T202/Y204) were purchased from Santa Cruz Biotechnology Inc. Anti-ZO-1 antibody was purchased from life Technologies, anti-TG2 antibody was from Thermo Fisher (Loughborough, UK).

### Cell culture conditions

Human CRC cell lines SW620 and HCT116, a kind gift from Dr Chris Tselepsis (University of Birmingham, UK) were cultured in a humidified atmosphere at 37ºC and 5% (v/v) CO_2_. All the cells were cultured in Dulbecco’s Modified Eagles Medium (DMEM) (Lonza, Slough, UK) containing 10% FBS (Thermo Fisher, Loughborough, UK), 1% (v/v) nonessential amino acids and penicillin/streptomycin (100U/ml and 100μg/ml respectively) used for experiments, unless otherwise indicated. Enrichment of spheroid cells with stem-like characteristics was performed by culturing cells on polyhema coated low attachment plates; cells were cultured in serum-free DMEM/F12 (1:1) (Lonza, Slough, UK) containing 2% (v/v) serum free supplement B27 (Thermo Fisher, Loughborough, UK), 20 ng/ml EGF, 0.4% bovine serum albumin and 4 μg/ml insulin. Spheroid cells were also cultured in humidified atmosphere at 37°C and 5% (v/v) CO2.

To collect the cells from the tumour spheroids the spheroids cultured in suspension for 7-10 days were collected by centrifugation at 300 ×g for 5 min, washed with sterile Phosphate buffered Saline (PBS), pH 7.4, and then the cells within the spheroid dispersed into single cells by treatment with pre-warmed 0.25% (w/v) trypsin in 2mM EDTA for 5 min at 37°C.

### Lentiviral Transduction

In order to silence TG2 in CRCs, lentiviral constructs containing wild-type shRNA that targets human TG2 was used to transduce the CRCs as previously described [43]. The CRCs were seeded into 6-well plates or 35 mm Petri dishes and allowed to reach 80% confluence. The complete growth media was then changed and renewed with 700 µl of fresh medium with 100 µl of lentiviral particles containing TG2 shRNA targeting human TG2 in each well and incubated for 24 h under standard cell culture conditions. A second infection was carried out the next day by adding 700 µl medium and 100 µl lentivirus per well and cells were incubated for another 24 h.shRNA sequences #1 5’-CCACCCACCATATTGTTTGAT- 3’ #2 5’-ACAGCAACCTTCTCATCGAGT-3’

### Cell treatment

CRCs were treated with the TG2 selective irreversible peptidomimetic cell-permeable inhibitor 1-155 [31] at 1 µM. CRCs were also treated with rhTGFβ1 (2.5 ng/ml) (R&D Systems, Oxford, UK) or mouse anti-human TGFβ neutralising antibody (R&D Systems, Oxford, UK). Control cells were treated with treatment vehicle DMSO (0.01%) or PBS.

### Western blotting

Cells were lysed in cell lysis buffer as described previously [42]. Equal amounts of protein were separated by SDS-PAGE and electroblotted onto nitrocellulose membranes as previously described [43, 44]. After incubation with primary and HRP-conjugated secondary antibodies, antigen-antibody complexes were visualized using enhanced chemiluminescence (Amersham Biosciences, Piscataway, USA). Densitometry was performed with ImageJ 1.49 for windows. Images were captured by a chemiluminescent image analyser Syngene G-box F3 (Cambridge, UK). The membranes were reprobed for GAPDH, an equal loading control used to normalize the signal obtained for target proteins. The ratios against the control treatment were analysed as described previously [31]. The intensity of the bands were normalised against GAPDH band.

### Dot Blotting

Equal numbers of viable cells from either the monolayer culture or the dispersed spheroids were seeded into 24-well plates (2.5×10^5^/well) in complete growth media for 4 h. After which the complete growth media was replaced with ITS (Sigma-Aldrich, Haverhill, UK) supplemented serum-free cell culture media. Cells were incubated for 18 h and the cell culture media collected and assayed by dot blotting to detect the presence of VEGF which was performed as described previously [44].

### Matrigel Cord formation assay

Endothelial cord formation was measured by a Matrigel endothelial cord formation assay as described by Wang *et al* (2013) [42]. 96 well plates were coated with Matrigel (Corning, Loughborough, UK). Human Umbilical Vein Endothelial Cells HUVECs (Promocell, Heidelberg, Germany) were seeded 1.5×10^5^ cells/well onto matrigel-coated wells and treated with conditioned media from spheroid cells or non-conditioned control media with or without rhVEGF as positive and negative controls.

### Cell Invasion Assay

Invasion assays were performed on serum-starved cells cultured in serum-free ITS (Insulin transferrin sodium selenite media supplement- Sigma Aldrich, UK) containing media for 16 h prior to seeding 1.0×10^5^ cells/100μl in same media onto the collagen IV-coated chamber Transwell inserts of a 24-well plate. 600 μl of 10% serum containing complete media were added to the accompanying 24-well plate of the chamber, and cells incubated for 24 h under cell culture conditions to allow for invasion. Following incubation, the Transwell inserts were removed from the plates and the media was discarded and non-migrated cells were removed. The cells that were embedded in the Transwell insert were fixed in 70% (v/v) ethanol for 10 min. After this the inserts and the Transwell membrane were allowed to dry. 0.2% crystal violet was used to stain the cells at room temperature [45]. Following staining, the inserts were removed, washed gently 3 times with PBS to remove the excess crystal violet, and the Transwell membranes were allowed to dry. The invasive cells were viewed underneath an inverted microscope and cell count was made under 10× objective. Images of the cells were also captured.

### Cell Viability Assay

XTT was used to measure cell viability of monolayer cells or cells collected from dispersed spheroids as described previously [23].

### Immunophenotyping by Flow Cytometry

Adherent monolayer cells and cells derived from tumour spheres were collected by trypsinisation and suspended in PBS containing 3% bovine serum albumin (BSA) (Sigma-Aldrich, UK). The cells were then initially blocked with 10% (v/v) normal human immunoglobulin (Ig) (Grifols, Cambridge, UK) for 30 min at 4 °C, after which cells were incubated with phycoerythrin-(PE) conjugated mouse anti-CD44 (Immunotools, Friesoythe, Germany) (1:100 dilution) in 3% (w/v) BSA in PBS, pH 7.4, for 2 h at 4ºC. Non-specific fluorescence was determined by incubating cells with isotype-matched control phycoerythrin-conjugated antibodies IgG2a (Immunotools, Friesoythe, Germany). Cells were then washed 4 times by centrifugation at 400 ×g in 3% (w/v) BSA in PBS, pH 7.4. Immunoreactivity for each CD44 was assessed by flow cytometry using a Beckman Coulter FC500 flow cytometer and data were analysed using Flowing Software 2.5.1 analysis Software.

### Statistical Analyses

Data were expressed as mean ± S.D. The data shown are derived from a representative experiment undertaken in triplicate (unless otherwise stated). Comparisons among different groups were performed by either t-test or analysis of variance using one-way ANOVA using the GraphPad Instat software package. Significant differences between control and treatment groups were analysed by Bonferroni’s Multiple Comparison Test. Statistical significant difference between data sets as defined in the text by p<0.05 (two-sided).

## SUPPLEMENTARY MATERIALS AND FIGURES



## Abbreviations

CRCs: Colorectal cancer
CSC: Cancer Stem cells
EMT: Epithelial-Mesenchymal Transition
ERK: Extracellular signal regulated kinase
HUVEC: Human Umbilical Vein Endothelial Cells
MAPK: Mitogen-activated protein kinases
PE: phycoerythrin
TG2: Tissue transglutaminase
TGFβ1: Transforming growth factor β1
VEGF: Vascular endothelial growth factor
XTT: Sodium 3’-[1-(phenylamino-carbonyl)-3,4-tetrazolium]-bis (4-methoxy-6-nitro) benzene sulfonic acid hydrate

## References

[1] Yadav AK , Desai NS . Cancer Stem Cells: Acquisition, Characteristics, Therapeutic Implications, Targeting Strategies and Future Prospects. Stem Cell Rev. 2019; 15:331–55. 10.1007/s12015-019-09887-2. . 30993589

[2] Wilson BJ , Schatton T , Frank MH , Frank NY Colorectal Cancer Stem Cells: Biology and Therapeutic Implications. Curr Colorectal Cancer Rep. 2011; 7:128-135. 10.1007/s11888-011-0093-2. . 21552371 PMC3087297

[3] Zhou Y , Xia L , Wang H , Oyang L , Su M , Liu Q , Lin J , Tan S , Tian Y , Liao Q , Cao D . Cancer stem cells in progression of colorectal cancer. Oncotarget. 2017; 9:33403–15. 10.18632/oncotarget.23607. . 30279970 PMC6161799

[4] Wei B , Han XY , Qi CL , Zhang S , Zheng ZH , Huang Y , Chen TF , Wei HB . Coaction of spheroid-derived stem-like cells and endothelial progenitor cells promotes development of colon cancer. PLoS One. 2012; 7:e39069. 10.1371/journal.pone.0039069. . 22745705 PMC3383752

[5] Kalluri R , Weinberg RA . The basics of epithelial-mesenchymal transition. J Clin Invest. 2009; 119:1420-1428. 10.1172/JCI39104. . 19487818 PMC2689101

[6] Mani SA , Guo W , Liao MJ , Eaton EN , Ayyanan A , Zhou AY , Brooks M , Reinhard F , Zhang CC , Shipitsin M , Campbell LL , Polyak K , Brisken C , et al. The epithelial-mesenchymal transition generates cells with properties of stem cells. Cell. 2008; 133:704–15. 10.1016/j.cell.2008.03.027. . 18485877 PMC2728032

[7] Hao Y , Baker D , Ten Dijke P . TGF-β-Mediated Epithelial-Mesenchymal Transition and Cancer Metastasis. Int J Mol Sci. 2019; 20:E2767. 10.3390/ijms20112767. . 31195692 PMC6600375

[8] Wang Z , Griffin M . TG2, a novel extracellular protein with multiple functions. Amino Acids. 2012; 42:939-949. 10.1007/s00726-011-1008-x. . 21818567

[9] Eckert RL , Kaartinen MT , Nurminskaya M , Belkin AM , Colak G , Johnson GV , Mehta K . Transglutaminase regulation of cell function. Physiol Rev. 2014; 94:383–417. 10.1152/physrev.00019.2013. . 24692352 PMC4044299

[10] Wang Z , Stuckey DJ , Murdoch CE , Camelliti P , Lip GY H, Griffin M . Cardiac fibrosis can be attenuated by blocking the activity of transglutaminase 2 using a selective small-molecule inhibitor. Cell Death Dis. 2018; 9:613. 10.1038/s41419-018-0573-2. . 29795262 PMC5966415

[11] Cao L , Shao M , Schilder J , Guise T , Mohammad KS , Matei D . Tissue transglutaminase links TGF-beta, epithelial to mesenchymal transition and a stem cell phenotype in ovarian cancer. Oncogene. 2012; 31:2521-2534. 10.1038/onc.2011.429. . 21963846

[12] Kumar A , Gao H , Xu J , Reuben J , Yu D , Mehta K . Evidence that aberrant expression of tissue transglutaminase promotes stem cell characteristics in mammary epithelial cells. PLoS One. 2011;6:e20701. 10.1371/journal.pone.0020701. . 21687668 PMC3110765

[13] Fisher ML , Keillor JW , Xu W , Eckert RL , Kerr C . Transglutaminase Is Required for Epidermal Squamous Cell Carcinoma Stem Cell Survival. Molecular Cancer Research. Mol Cancer Res. 2015; 13:1083–94. 10.1158/1541-7786.MCR-14-0685-T. . 25934691 PMC4504806

[14] Adhikary G , Grun D , Alexander HR , Friedberg JS , Xu W , Keillor JW , Kandasamy S , Eckert RL . Transglutaminase is a mesothelioma cancer stem cell survival protein that is required for tumor formation. Oncotarget. 2018; 9:34495-34505. 10.18632/oncotarget.26130. . 30349644 PMC6195372

[15] Condello S , Morgan CA , Nagdas S , Cao L , Turek J , Hurley TD , Matei D . beta-Catenin-regulated ALDH1A1 is a target in ovarian cancer spheroids. Oncogene. 2015; 34:2297-2308. 10.1038/onc.2014.178. . 24954508 PMC4275429

[16] Pasche B , Mulcahy M , Benson AB . Molecular markers in prognosis of colorectal cancer and prediction of response to treatment. Best Practice & Research in Clinical Gastroenterology. Best Pract Res Clin Gastroenterol. 2002; 16:331-345. 10.1053/bega.2002.0289. . 11969242

[17] Kiyonari H , Kaneko M , Abe S , Aizawa S . Three inhibitors of FGF receptor, ERK, and GSK3 establishes germline-competent embryonic stem cells of C57BL/6N mouse strain with high efficiency and stability. Genesis. 2010; 48:317-27. 10.1002/dvg.20614. . 20162675

[18] Ayinde O , Wang Z , Griffin M . Tissue transglutaminase induces Epithelial-Mesenchymal-Transition and the acquisition of stem cell like characteristics in colorectal cancer cells. Oncotarget. 2017; 8:20025-20041. 10.18632/oncotarget.15370. . 28223538 PMC5386741

[19] Todaro M , Alea MP , Di Stefano AB , Cammareri P , Vermeulen L , Iovino F , Tripodo C , Russo A , Gulotta G , Medema JP , Stassi G . Colon cancer stem cells dictate tumor growth and resist cell death by production of interleukin-4. Cell Stem Cell. 2007; 1:389-402. 10.1016/j.stem.2007.08.001. . 18371377

[20] Han XY , Wei B , Fang JF , Zhang S , Zhang FC , Zhang HB , Lan TY , Lu HQ , Wei HB . Epithelial-mesenchymal transition associates with maintenance of stemness in spheroid-derived stem-like colon cancer cells. PLoS One. 2013; 8:e73341. 10.1371/journal.pone.0073341. . 24039918 PMC3767831

[21] Miki T , Yasuda SY , Kahn M . Wnt/beta-catenin signaling in embryonic stem cell self-renewal and somatic cell reprogramming. Stem Cell Rev. 2011; 7:836-846. 10.1007/s12015-011-9275-1. . 21603945

[22] Fisher ML , Adhikary G , Xu W , Kerr C , Keillor JW , Eckert RL . Type II transglutaminase stimulates epidermal cancer stem cell epithelial-mesenchymal transition. Oncotarget. 2015; 6:20525-20539. 10.18632/oncotarget.3890. . 25971211 PMC4653023

[23] Cao L , Zhou Y , Zhai B , Liao J , Xu W , Zhang R , Li J , Zhang Y , Chen L , Qian H , Wu M , Yin Z . Sphere-forming cell subpopulations with cancer stem cell properties in human hepatoma cell lines. BMC Gastroenterol. 2011; 11:71. 10.1186/1471-230X-11-71. . 21669008 PMC3136412

[24] Kumar A , Xu J , Sung B , Kumar S , Yu D , Aggarwal BB , Mehta K . Evidence that GTP-binding domain but not catalytic domain of transglutaminase 2 is essential for epithelial-to-mesenchymal transition in mammary epithelial cells. Breast Cancer Res. 2012; 14:R4. 10.1186/bcr3085. . 22225906 PMC3496119

[25] Ling GQ , Chen DB , Wang BQ , Zhang LS . Expression of the pluripotency markers Oct3/4, Nanog and Sox2 in human breast cancer cell lines. Oncol Lett. 2012; 4:1264-1268. 10.3892/ol.2012.916. . 23197999 PMC3506717

[26] Kang S , Oh SC , Min BW , Lee DH . Transglutaminase 2 Regulates Self-renewal and Stem Cell Marker of Human Colorectal Cancer Stem Cells. Anticancer Res. 2018; 38:787–94. 10.21873/anticanres.12285. . 29374703

[27] Fan F , Samuel S , Evans KW , Lu J , Xia L , Zhou Y , Sceusi E , Tozzi F , Ye XC , Mani SA , Ellis LM . Overexpression of snail induces epithelial-mesenchymal transition and a cancer stem cell-like phenotype in human colorectal cancer cells. Cancer Med. 2012; 1:5-16. 10.1002/cam4.4. . 23342249 PMC3544430

[28] Polyak K , Weinberg RA . Transitions between epithelial and mesenchymal states: acquisition of malignant and stem cell traits. Nat Rev Cancer. 2009. 9:265–73. 10.1038/nrc2620. . 19262571

[29] Eckert RL , Fisher ML , Grun D, Adhikary G , Xu W , Kerr C . Transglutaminase is a tumor cell and cancer stem cell survival factor. Mol Carcinog. 2015. 54:947–58. 10.1002/mc.22375. . 26258961 PMC4752121

[30] Agnihotri N , Kumar S , Mehta K . Tissue transglutaminase as a central mediator in inflammation-i nduced progression of breast cancer. Breast Cancer Res. 2013; 15:202. 10.1186/bcr3371. . 23673317 PMC3745644

[31] Badarau E , Wang Z , Rathbone DL , Costanzi A , Thibault T , Murdoch CE , El Alaoui S , Bartkeviciute M , Griffin M . Development of Potent and Selective Tissue Transglutaminase Inhibitors: Their Effect on TG2 Function and Application in Pathological Conditions. Chem Biol. 2015; 22:1347-1361. 10.1016/j.chembiol.2015.08.013. . 26456735

[32] Novellasdemunt L , Antas P , Li VS . Targeting Wnt signaling in colorectal cancer. A Review in the Theme: Cell Signaling: Proteins, Pathways and Mechanisms. Am J Physiol Cell Physiol. 2015; 309:C511-521. 10.1152/ajpcell.00117.2015. . 26289750 PMC4609654

[33] Condello S , Sima L , Ivan C , Cardenas H , Schiltz G , Mishra RK , Matei D . Tissue Tranglutaminase Regulates Interactions between Ovarian Cancer Stem Cells and the Tumor Niche. Cancer Res. 2018; 78:2990-3001. 10.1158/0008-5472.CAN-17-2319. . 29510995 PMC5984683

[34] Lemieux E , Cagnol S , Beaudry K , Carrier J , Rivard N . Oncogenic KRAS signalling promotes the Wnt/beta-catenin pathway through LRP6 in colorectal cancer. Oncogene. 2015; 34:4914-4927. 10.1038/onc.2014.416. . 25500543 PMC4687460

[35] Horst D , Chen J , Morikawa T , Ogino S , Kirchner T , Shivdasani RA . Differential WNT activity in colorectal cancer confers limited tumorigenic potential and is regulated by MAPK signaling. Cancer Res. 2012; 72:1547-1556. 10.1158/0008-5472.CAN-11-3222. . 22318865 PMC3571091

[36] Shtutman M , Zhurinsky J , Simcha I , Albanese C , D' Amico M , Pestell R , Ben-Ze'ev A. The cyclin D1 gene is a target of the beta-catenin/LEF-1 pathway. Proc Natl Acad Sci U S A. 1999; 96:5522-5527. 10.1073/pnas.96.10.5522. . 10318916 PMC21892

[37] Kafri P , Hasenson SE , Kanter I , Sheinberger J , Kinor N , Yunger S , Shav-Tal Y . Quantifying beta-catenin subcellular dynamics and cyclin D1 mRNA transcription during Wnt signaling in single living cells. Elife. 2016; 5:e16748. 10.7554/eLife.16748. . 27879202 PMC5161448

[38] Rojas-Puentes L , Cardona AF , Carranza H , Vargas C , Jaramillo LF , Zea D , Cetina L , Wills B , Ruiz-Garcia E , Arrieta O . Epithelial-mesenchymal transition, proliferation, and angiogenesis in locally advanced cervical cancer treated with chemoradiotherapy. Cancer Med. 2016; 5:1989-1999. 10.1002/cam4.751. . 27230280 PMC4884920

[39] Fantozzi A , Gruber DC , Pisarsky L , Heck C , Kunita A , Yilmaz M , Meyer-Schaller N , Cornille K , Hopfer U , Bentires-Alj M , Christofori G . VEGF-mediated angiogenesis links EMT-induced cancer stemness to tumor initiation. Cancer Res. 2014; 74:1566-1575. 10.1158/0008-5472.CAN-13-1641. . 24413534

[40] Zhang X , Gaspard JP , Chung DC . Regulation of vascular endothelial growth factor by the Wnt and K-ras pathways in colonic neoplasia. Cancer Res. 2001; 61:6050-6054. . 11507052

[41] Easwaran V , Lee SH , Inge L , Guo L , Goldbeck C , Garrett E , Wiesmann M , Garcia PD , Fuller JH , Chan V , Randazzo F , Gundel R , Warren RS , et al. beta-Catenin regulates vascular endothelial growth factor expression in colon cancer. Cancer Res. 2003; 63:3145–53. . 12810642

[42] Wang Z , Perez M , Caja S , Melino G , Johnson TS , Lindfors K , Griffin M . A novel extracellular role for tissue transglutaminase in matrix-bound VEGF-mediated angiogenesis. Cell Death Dis. 2013; 4:e808. 10.1038/cddis.2013.318. . 24052076 PMC3789176

[43] Wang Z , Griffin M . The role of TG2 in regulating S100A4-mediated mammary tumour cell migration. PLoS One. 2013; 8:e57017. 10.1371/journal.pone.0057017. . 23469180 PMC3585722

[44] Wang Z , Collighan RJ , Pytel K , Rathbone DL , Li X , Griffin M . Characterization of heparin-binding site of tissue transglutaminase: its importance in cell surface targeting, matrix deposition, and cell signaling. J Biol Chem. 2012. 287:13063–83. 10.1074/jbc.M111.294819. . 22298777 PMC3339925

[45] Kotsakis P , Wang Z , Collighan RJ , Griffin M . The role of tissue transglutaminase (TG2) in regulating the tumour progression of the mouse colon carcinoma CT26. Amino Acids. 2011; 41:909–21. 10.1007/s00726-010-0790-1. . 21046178

