# Supplementary material for: Transglutaminase 2 maintains a colorectal cancer stem phenotype by regulating epithelial-mesenchymal transition
Source: Oncotarget. 2019 Jul 16;10(44):4556–69. doi: 10.18632/oncotarget.27062 (PMC6642042; doi:10.18632/oncotarget.27062)
Supplement: Supplementary file 1 [file oncotarget-10-4556-s001.pdf]

Transglutaminase 2 maintains a colorectal cancer stem phenotype by regulating Epithelial-mesenchymal transition

SUPPLEMENTARY MATERIALS

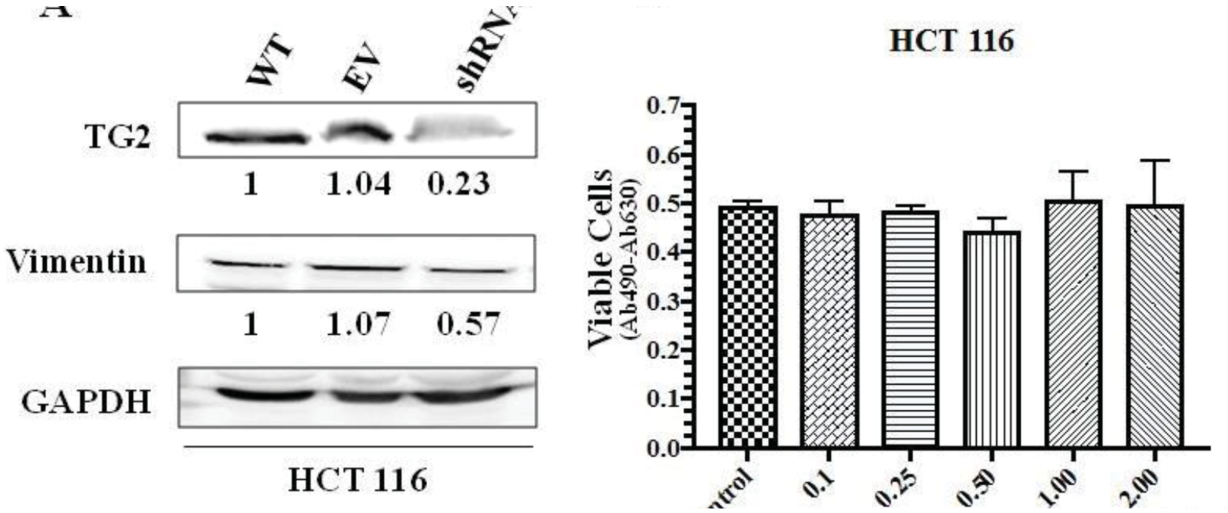

Supplementary Figure 1: Empty Vector transduction has comparable TG2 and EMT profile to wild-type cells. **(A)** Western blot detection of TG2 and vimentin in empty vector, TG2 shRNA, and wild-type HCT116 cells. Ratios indicate mean relative densitometry ratios of protein expression of the proteins against GAPDH. n=2. **(B)** TG2 inhibition does not affect cell viability in HCT116 cells as measured by XTT over a 48 h treatment period. n=3.
